# Treatment outcome and associated factors of pyogenic meningitis among children at Hawassa University comprehensive specialized hospital: a retrospective cross-sectional study

**DOI:** 10.1186/s12887-026-07092-y

**Published:** 2026-06-18

**Authors:** Anteneh D. Teklemariam, Bezawit D. Hailu, Alazar M. Tesfaye, Tinbit E. Woldegebriel, Yabets A. Mamo, Bereket A. Tegene, Dawit A. Bitewa

**Affiliations:** 1 Hawassa University Comprehensive Specialized Hospital, Hawassa, Ethiopia; 2 Faculty of Medical Science, Jimma University Institute of Health, Jimma, Ethiopia; 3 Tikur Anbessa Specialised Hospital, Addis Ababa, Ethiopia; 4 College of Health Science, Debre Markos University, Debre Markos, Ethiopia

**Keywords:** Pyogenic meningitis, Children, Outcome, Ethiopia

## Abstract

**Background:**

In Ethiopia, little is known about short-term treatment outcomes of bacterial meningitis and its associated factors among hospitalized children. This study aimed to assess the magnitude and predictors of unfavourable treatment outcome among these group.

**Methods:**

A five-year retrospective study was conducted from September 2020 to August 2024 at Hawassa University Comprehensive Specialized Hospital. All children aged 1 month–14 years diagnosed with pyogenic meningitis and admitted to the pediatric ward or emergency department were included. Data were collected from medical charts using a structured tool. Statistical analyses were performed using SPSS v26, with binary logistic regression to identify factors associated with treatment outcomes. Statistical significance was declared at *p* < 0.05.

**Result:**

Among 203 patients, 67 (33%; 95% CI 26%, 40%) had unfavourable outcomes: 29 (14.3%) died, 16 (7.9%) developed acute neurologic complications, and 22 (10.8%) were discharged against medical advice. Being male [AOR: 2.87; 95% CI: 1.14, 7.17], malnourished [AOR: 5.4; 95% CI: 2.28, 12.8], incomplete vaccination [AOR: 4.64; 95%CI: 1.71, 12.5], developing aspiration pneumonia [AOR: 4.5; 95% CI: 1.1, 17.5], comatose upon presentation [AOR: 4.6; 95% CI: 1.42, 14.9] were significantly associated with unfavourable treatment outcomes.

**Conclusion:**

One-third of children hospitalized with pyogenic meningitis experienced unfavourable outcomes. Malnutrition, incomplete immunization, coma on presentation, and aspiration pneumonia were key predictors. Strengthening nutrition programs and immunization coverage may reduce morbidity and mortality.

**Supplementary Information:**

The online version contains supplementary material available at https://doi.org/10.1186/s12887-026-07092-y.

## Introduction

Meningitis is an acute inflammation of the meninges involving the subarachnoid space and cerebrospinal fluid. In children, acute bacterial meningitis (ABM) remains one of the most severe infectious diseases, associated with high mortality and a substantial risk of neurological complications despite advances in antimicrobial therapy [1–3]. Definitive diagnosis typically relies on cerebrospinal fluid (CSF) analysis and culture; however, in many low-resource settings, limited diagnostic capacity necessitates reliance on clinical diagnosis and empiric treatment [4–6].

Globally, the burden of bacterial meningitis remains substantial, with the highest incidence and mortality observed among young children in low- and middle-income countries. Sub-Saharan Africa, including countries within and adjacent to the African meningitis belt, continues to experience a disproportionate burden due to delayed presentation, limited access to advanced diagnostics, and variability in quality of care [2, 7, 8]. Survivors frequently experience short-term complications such as seizures, focal neurological deficits, hydrocephalus, and cerebral edema, which contribute significantly to in-hospital morbidity and mortality [3, 5, 9].

Pediatric bacterial meningitis is largely preventable through routine childhood immunization against common pathogens such as *Haemophilus influenzae* type b, *Streptococcus pneumoniae*, and *Neisseria meningitidis* [7, 9]. Although Ethiopia has incorporated these vaccines into the national Expanded Programme on Immunization, vaccination coverage remains uneven, particularly in rural and underserved populations [8, 10]. Incomplete immunization continues to expose children to severe and preventable infections, potentially influencing disease severity at presentation and short-term treatment outcomes [11, 12].

In Ethiopia, bacterial meningitis remains a significant cause of pediatric hospitalization and mortality. Hospital-based studies from different regions of the country have reported considerable case fatality rates and variable clinical outcomes, highlighting ongoing challenges in early recognition, timely treatment, and supportive care [8, 10, 11, 13, 14]. However, available evidence is inconsistent across settings, and detailed analyses focusing specifically on treatment outcomes and associated factors among hospitalized children remain limited.

Focusing on treatment outcomes, including in-hospital mortality, acute neurological complications, and discharge against medical advice, is particularly relevant in resource-limited settings where early outcomes reflect both disease severity and the effectiveness of acute clinical management [5, 11, 15]. Identifying factors associated with unfavourable treatment outcomes can inform targeted interventions to improve early case management, reduce preventable deaths, and optimize use of limited health-care resources.

Therefore, this study aimed to assess the magnitude of unfavourable treatment outcomes and identify associated factors among children hospitalized with pyogenic meningitis at Hawassa University Comprehensive Specialized Hospital, southern Ethiopia.

## Method and material

### Study setting, study design and study period

The study was conducted at HUCSH, Hawassa City, Ethiopia, which is the capital of Sidama Region, located 275 km south of Addis Ababa (capital of Ethiopia). It was hospital based five-year retrospective study conducted from Sept 2020 to Aug 2024.

### Study participants

All paediatric patients from age 1month to 14 years hospitalized with a presumptive (physician’s clinical or laboratory-based) diagnosis of community acquired acute bacterial meningitis whose medical charts of patients fulfil the inclusion and exclusion criteria. This age range reflects the upper age limit for admission to the pediatric ward at HUCSH and is consistent with the definition of pediatric populations in similar Ethiopian studies.

### Sample size calculation and sampling

All eligible pediatric cases of pyogenic meningitis admitted during the study period were included. As this was a retrospective chart review, the sample size was determined by the number of available cases that met the inclusion criteria, resulting in a final sample size of 203 patients.

### Dependent and independent variables

The Short term treatment outcome was the dependent variable and the rest including age, sex, socioeconomic status, duration of illness before presentation, Severity of illness at presentation (loss of consciousness, seizure), nutrition status, immunization status, antibiotic regimen, corticosteroid administration, ICP management, anticonvulsants use were independent variables.

### Inclusion and exclusion criteria

All paediatric meningitis cases from the age of one month to fourteen years who were admitted to HUCSH from Sept 2020 to Aug 2024, and whose charts contained comprehensive medical information were included. Patients with incomplete or poor medical records, initial diagnosis of meningitis changed or if there was diagnosis of concomitant tuberculous meningitis were excluded.

### Operational definition

#### Bacterial meningitis

Bacterial meningitis was defined as a diagnosis made by the treating physician based on clinical features consistent with meningitis (such as fever, neck stiffness, altered consciousness, or seizures), with or without supportive cerebrospinal fluid (CSF) findings, in children aged 0–14 years admitted to HUCSH during the study period.

#### Antibiotic-regimen change

Defined as a change in empirical antibiotics within 2–3 days in cases where the patient was not improving with initial empiric antibiotics.

#### Treatment outcome

Defined as the clinical status of the child at hospital discharge.

#### Favourable outcome

Improvement and discharge without acute neurological complications.

#### Unfavourable outcome

Included in-hospital death, development of acute neurological complications (such as hydrocephalus, brain abscess, focal neurological deficits), or discharge against medical advice (DAMA).

#### Immunization status

Determined from vaccination cards or caregiver reports documented in medical records. Children were classified as fully vaccinated, partially vaccinated, unvaccinated, or unknown based on the national Expanded Programme on Immunization schedule.

#### Nutritional status

Assessed using anthropometric measurements recorded at admission. For children under five years, weight-for-height Z-scores (WHZ) were used based on WHO Child Growth Standards. For children aged five years and above, BMI-for-age Z-scores were used based on WHO reference standards. Moderate malnutrition was defined as Z-score between − 3 and − 2 SD, and severe malnutrition as Z-score < − 3 SD.

#### Comorbidities

Defined as additional medical conditions present at admission that could influence clinical outcomes. These included malaria, aspiration pneumonia, pneumonia, hospital-acquired infections, sepsis, hypocalcaemia, iron-deficiency anemia, otitis media, rickets, tonsillopharyngitis, urinary tract infection, traumatic brain injury, and pertussis.

### Data collection

Data were collected using structured standard checklist that is adapted from earlier studies in the field or scientific best practices, with some modifications made by the principal investigator consulting an experienced expert clinician (paediatric neurologist) after a thorough review of pertinent literature. Trained data collectors (two medical interns) collected the data and the principal investigator followed and monitored thoroughly the process of data collection and data is checked for completeness daily.

All admitted children underwent a detailed history, physical examination and a lumbar puncture where not contraindicated. Due to low culture positivity, likely related to prior antibiotic exposure, most cases were clinically diagnosed. All suspected cases were treated empirically according to standard hospital protocol without waiting for laboratory confirmation. Children were discharged when afebrile and after completing their antibiotics. Follow up in the study was during the hospitalization days of the patients.

### Statistical analysis

Data extracted from patient medical charts were entered into Microsoft Excel, coded and cleaned, and then exported to SPSS version 26 for analysis. Descriptive statistics were used to summarize sociodemographic, clinical, and management characteristics of the study participants. Bivariable logistic regression analysis was performed to assess associations between independent variables and treatment outcome. Variables with a p-value < 0.25 in bivariable analysis were entered into a multivariable logistic regression model to identify independent predictors of unfavourable outcome. Model fitness was assessed using the Hosmer–Lemeshow goodness-of-fit test, and statistical significance was declared at *p* < 0.05.

### Ethical considerations

Ethical approval was obtained from the Institutional Review Board of Hawassa University College of Medicine and Health Sciences (Ref No: IRB/373/16). Permission was also obtained from the Department of Pediatrics and Child Health. The requirement for informed consent was waived as only chart data were used. Confidentiality was strictly maintained.

## Results

### Sociodemographic, clinical, nutritional, and immunization characteristics

A total of 203 children were included in the study. 94(46.3%) Were aged five years or below, with a median age of 5 years (Interquartile range IQR = 8). 132 (65%) of the children were male, and 146 (71.9%) came from rural areas. Regarding vaccination status, 131 (64.5%) were fully vaccinated, 39 (19.2%) were partially vaccinated, 26 (12.8%) were unvaccinated, and 7 (3.4%) had unknown vaccination status. Eighteen children (8.9%) were malnourished, while 38 (18.7%) had moderate malnutrition. Comorbid conditions were identified in 110 children (54.2%), with malaria being the most common (46/110, 41.8%), followed by aspiration pneumonia (29/110, 27.6%). Most children presented late to the hospital, with 157 (77.3%) arriving after three or more days of illness. The mean duration of illness prior to admission was 6 days (SD 4.2) (Table 1).

**Table 1:** Sociodemographic, Clinical, nutritional, and immunization characteristics of the study participants admitted to HUCSH, 2024

Variables	Categories	Frequency	Percentage
Age in years	< 5	94	46.3
	6–10	70	34.5
	> 10	39	19.2
Sex	Male	132	65.0
	Female	71	35.0
Residence	Rural	146	71.9
	Urban	57	28.1
Immunization status	Fully vaccinated	131	64.5
	Partially vaccinated	39	19.2
	Unknown	7	3.4
	Unvaccinated	26	12.8
Nutritional status	Severe Malnourished	18	8.9
	Moderate malnutrition	38	18.7
	Normal	147	72.4
Comorbidity	Yes	110	54.2
	No	93	45.8
Type of comorbidity(*n* = 110)	Malaria	46	41.8
	Pneumonia	26	23.6
	Sepsis	10	9.1
	Other¥	24	21.8
	Hospital Acquired Infection (HAI)	13	11.8
	Aspiration pneumonia	29	27.6
Prior antibiotic use	No	87	42.9
	Yes	116	57.1
Duration of illness in days	< 3	46	22.7
	*≥* 3	157	77.3

¥ (Hypocalcemia, IDA, Otitis media, Rickets, tonsillopharyngitis, UTI, TBI, Pertusis)

### Clinical presentation

**Fig. 1:**
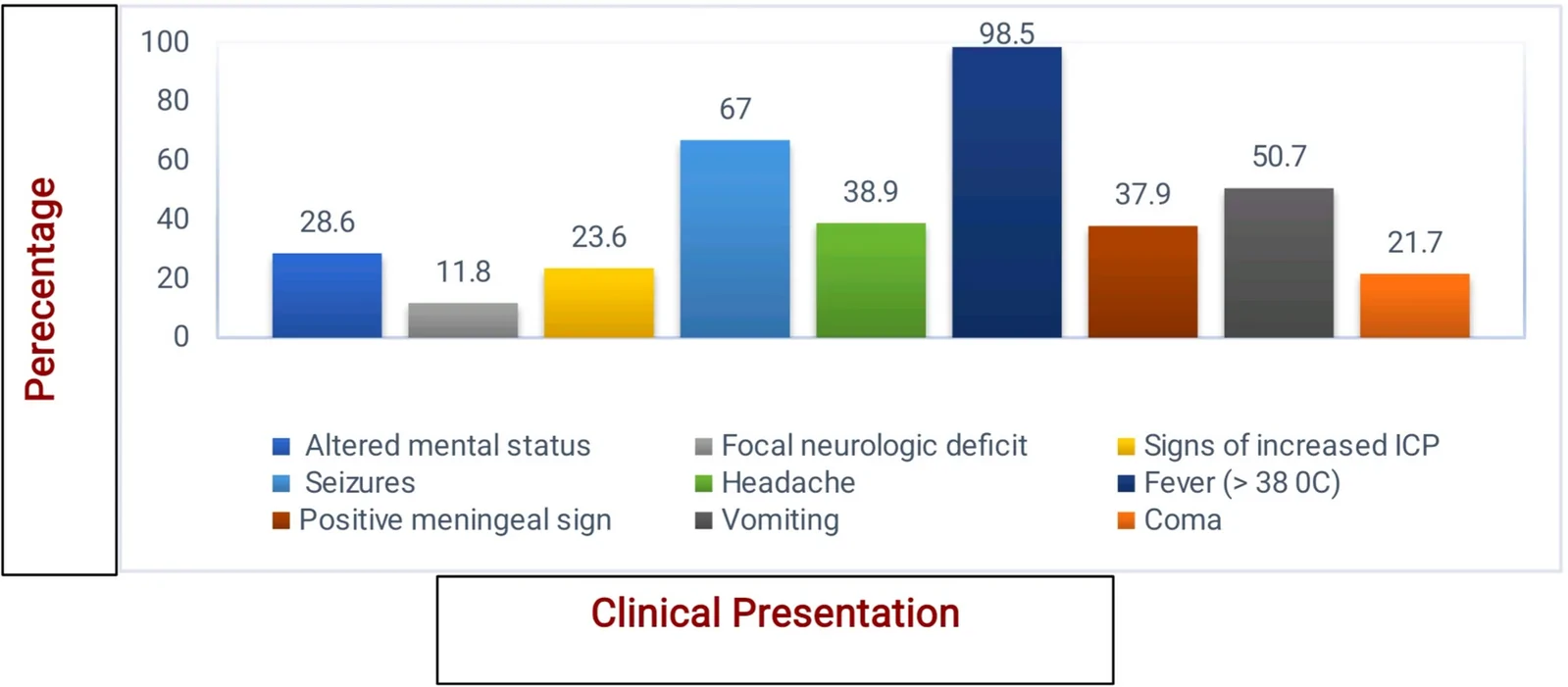
Clinical presentation of pyogenic meningitis among children admitted to HUCSH, 2024

The most common presenting symptom was fever, reported in 200 (98.5%) children. Seizures were observed in 136 (67.0%) patients, while vomiting occurred in 103 (50.7%). Indicators of severe neurological involvement were present in a notable proportion of children, including coma at presentation in 44 (21.7%) and focal neurological deficits in 24 (11.8%). Other presenting symptoms are summarized in (Fig. 1).

### Investigations and management

Over one-thirds of children 77 (37.9%) had cerebrospinal fluid analysis done, and 37 (18.2%) CSF cultures were done. Neither gram stain nor culture reports showed any organism. Neuroimaging was done for 93 (45.8%) of children. Neuroimaging was not routinely performed at initial diagnosis; it was conducted selectively in children with suspected complications such as persistent altered consciousness, focal neurological deficits, or signs of increased intracranial pressure. Among those who underwent imaging, cerebral edema was the main finding 39 (41.9%), followed by communicating hydrocephalus 19 (20.4%) and meningeal enhancement 17 (18.3%). Vancomycin 193 (95.1%) is a widely used antibiotic, followed by Ceftriaxone 185 (91.1%), while meropenem 2 (1.0%) and penicillin 6 (3.0%) are less commonly used antibiotics (Table 2).

**Table 2:** Investigations and management of children with pyogenic meningitis admitted to HUCSH, 2024

Variables	Categories	Frequency	Percentage
CSF analysis	Yes	77	37.9
	No	126	62.1
CSF culture	Yes	37	18.2
	No	166	81.8
Neuro imaging	Yes	93	45.8
	No	110	54.2
Imaging findings (*n* = 93)	Meningeal enhancement	17	18.3
	Cerebral edema	39	41.9
	Hydrocephalus	19	20.4
	Subdural effusion edema	4	4.3
	Focal infarction	13	14.0
	Others	3	3.2
	Normal	31	33.3
Medication used	Ceftriaxone	185	91.1
	Vancomycin	193	95.1
	Ceftazidime	27	13.3
	Cefepime	7	3.6
	Meropenem	2	1.0
	Penicillin	6	3.0
	Metronidazole	26	12.8
Corticosteroid	No	57	28.1
	Yes	146	71.9
ICP management	No	114	56.2
	Yes	89	43.8
Anticonvulsant	No	79	38.9
	Yes	124	61.1

### Treatment outcomes

**Fig. 2:**
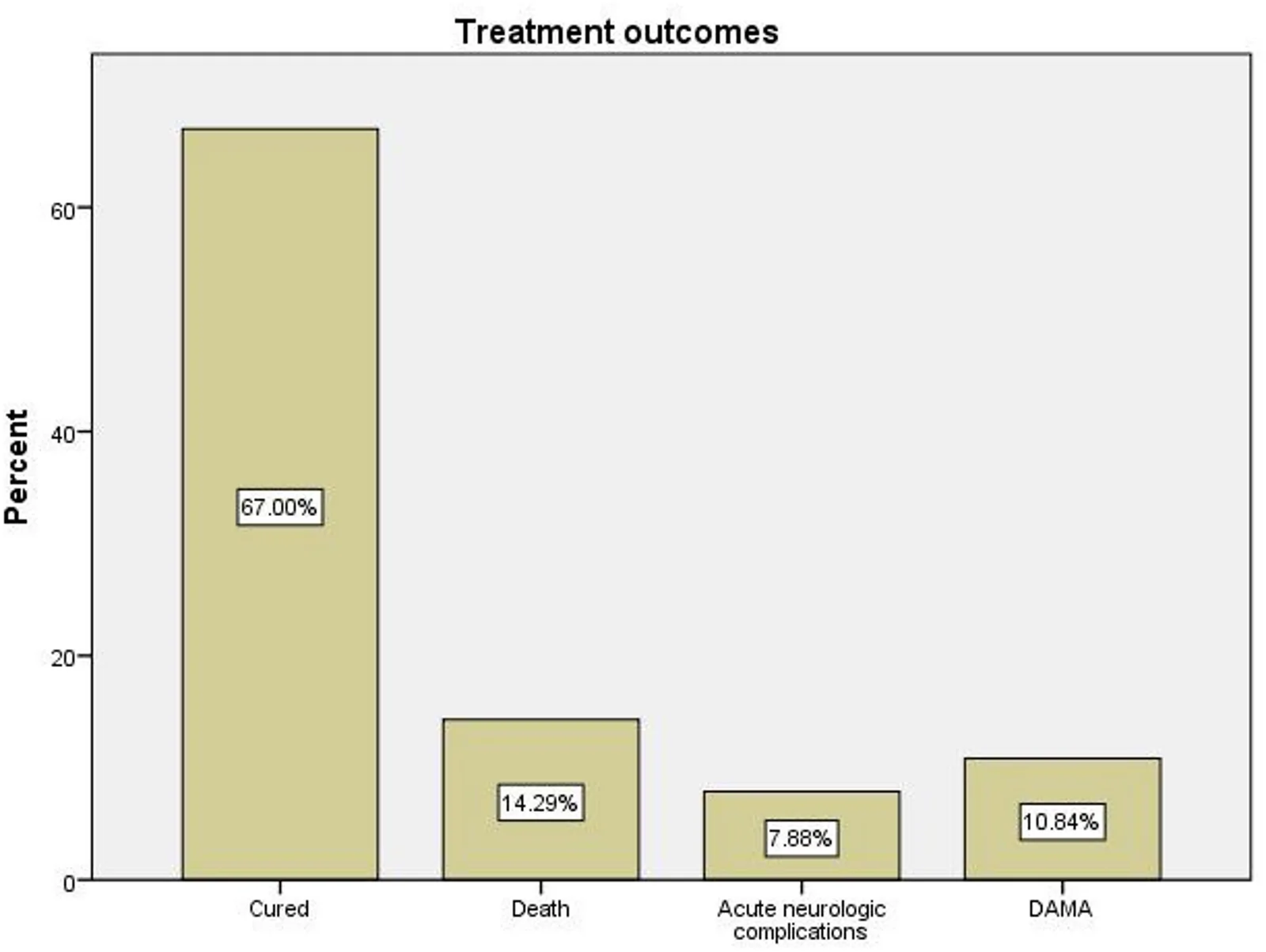
Treatment outcomes of children diagnosed with pyogenic meningitis at HUCSH, 2024

The study found that 67 (33%; 95% CI; 26%, 40%) children had unfavorable outcome. Among unfavourable treatment outcomes, 29 (14.3%) died, 16 (7.9%) had developed acute neurologic complications, and 22 (10.8%) were discharged against medical advice (Fig. 2).

The study found a significant association between neuroimaging finding of cerebral edema and unfavourable treatment outcome (66.7% vs. 27.8%, *p* < 0.001). Similarly, there is significant association b/n finding of having focal infarction and unfavourable treatment outcome (69.2% vs. 40%, *p* = 0.049). The study did not find statistically significant association between CSF WBC count (> 10cell) and unfavourable outcome (29.4% vs. 15.8%, *p* = 0.165).

### Factors associated with treatment outcomes

In bivariable logistic regression analysis, sex, residence, duration of illness, vaccination status, nutritional status, comorbidity, aspiration pneumonia, prior antibiotic use, signs of increased intracranial pressure, corticosteroid use, and coma at presentation were associated with unfavourable outcome at *p* < 0.25 and were entered into multivariable analysis.

In the multivariable model, nutritional status, vaccination status, aspiration pneumonia, and coma at presentation remained independently associated with unfavourable outcome. Malnourished children had higher odds of unfavourable outcome compared to well-nourished children (AOR = 5.4; 95% CI: 2.28–12.8). Children with incomplete or unknown vaccination status had increased odds of unfavourable outcome (AOR = 4.64; 95% CI: 1.71–12.5). Aspiration pneumonia (AOR = 4.5; 95% CI: 1.1–17.5) and coma at presentation (AOR = 4.6; 95% CI: 1.42–14.9) were also significant predictors (Table 3).

**Table 3:** Binary logistic analysis to identify factors associated with treatment outcomes of children with pyogenic meningitis admitted to HUCSH, 2024

Variables	Categories	Treatment outcome		COR (95%CI)	*p*-value	AOR (95%CI)
		unfavourable	unfavourable			
Age	*≤* 5	62(66.0)	32(34.0)	1		
	6–10	46(65.7)	24(34.3)	1.01(0.52,1.94)	0.974	
	> 10	28(71.8)	11(28.2)	0.76(0.33,1.72)	0.513	
Sex	Male	80(60.6)	52(39.4)	2.42(1.24,4.73)	0.008	2.87(1.14,7.17) *
	Female	56(78.9)	15(21.1)	1		1
Residence	Rural	89(61.0)	57(39.0)	3.01(1.40,6.43)	0.003	1.09(0.35,3.32)
	Urban	47(82.5)	10(17.5)	1		1
Duration of illness	< 3 days	37(80.4)	9(19.6)	1		1
	>=3 days	99(63.1)	58(36.9)	2.41(1.08,5.34)	0.028	1.40(0.33,5.86)
Fully vaccinated	Yes	105(80.2)	26(19.8)	1		1
	No	31(43.1)	41(56.9)	5.34(2.83,10.1)	< 0.001	4.64(1.71,12.5) **
Nutritional status	Malnourished	19(33.9)	37(66.1)	7.6(3.83,15.0)	< 0.001	5.4(2.28,12.8) **
	Normal	117(79.6)	30(20.4)	1		1
Commodity	Yes	65(59.1)	45(40.9)	2.23(1.21,4.11)	0.009	1.52(0.55,4.15)
	No	71(76.3)	22(23.7)	1		1
Aspiration pneumonia	Yes	6(20.7)	23(79.3)	11.3(4.3,29.6)	< 0.001	4.5(1.1,17.5) **
	No	130(74.7)	44(25.3)	1		1
Prior antibiotic use	Yes	70(60.3)	46(39.7)	1		1
	No	66(75.9)	21(24.1)	0.48(0.26,0.89)	0.02	0.45(0.18,1.12)
Sign of increased ICP	Yes	17(35.4)	31(64.6)	6.0(2.99,12.1)	< 0.001	2.12(0.70,6.39)
	No	119(76.8)	36(23.2)	1		1
Corticosteroid use	Yes	102(69.9)	44(30.1)	0.63(0.33,1.20)	0.164	1.15(0.43,3.05)
	No	34(59.6)	23(40.4)	1		1
Coma at presentation	Yes	15(34.1)	29(65.9)	6.1(2.99,12.67)	< 0.001	4.6(1.42,14.9) *
	No	121(76.1)	38(23.9)	1		1

*AOR* adjusted odds ratio, *COR* crude odds ratio, *ICP* intracranial pressure

*Significant at a *p*-value < 0.05 and ** significant at a *p*-value < 0.001 level

## Discussion

Acute bacterial meningitis remains a major cause of morbidity and mortality among children in low-resource settings despite improvements in vaccination coverage and antimicrobial therapy [16, 17]. In this study, nearly half(46.3%) of cases occurred in children aged five years or below, consistent with reports from Kandahar, Afghanistan (48.8%) [18] and Felege Hiwot Referral Hospital (65.3%) [11]. This age distribution reflects the increased vulnerability of younger children due to immature immune responses, higher exposure risk, and incomplete vaccination status in some settings [16].

A large proportion of children in our study were from rural areas (71.9%), Which is comparable to findings from studie conducted in other parts of the countr, such as Dilla (67.5%) [14] and Wolaita Sodo (81.7%) [10]. Rural residence in Ethiopia is often associated with delayed presentation, limited access to healthcare facilities, and late initiation of appropriate antimicrobial therapy. These factors likely contribute to advanced disease at admission and may partly explain the observed burden of unfavorable outcomes [19].

The overall proportion of favorable outcomes in this study was 77%, comparable to reports from Bahirdar (76%) [11] and Eastern Ethiopia (77%) [8], but lower than findings from India (89%) [20]. Differences in outcome across settings may reflect variation in healthcare access, early recognition, pathogen distribution, and availability of supportive care such as intensive monitoring and neurocritical support. Importantly, one-third of children in our cohort experienced an unfavorable outcome, underscoring that acute bacterial meningitis continues to pose a significant clinical challenge in this context [16, 17].

Four factors were independently associated with unfavorable outcomes: malnutrition, incomplete vaccination, aspiration pneumonia, and coma at presentation. Malnutrition increased the odds of Unfavourable outcome more than fivefold, consistent with findings from Wolaita Sodo [10] and Bangladesh [12]. The biological plausibility of this association is well established. Protein-energy malnutrition impairs cell-mediated immunity, reduces complement activity, and compromises mucosal and blood–brain barrier defenses, thereby increasing susceptibility to invasive infection and limiting the host’s ability to control systemic inflammation [21, 22]. In the context of meningitis, this may translate into higher bacterial burden, exaggerated inflammatory responses, and poorer neurological recovery.

Coma at presentation was also strongly associated with unfavorable outcome, in agreement with studies from Bahirdar [11], Hiwot Fana [8], and Madagascar [23]. Altered consciousness reflects advanced intracranial pathology, including cerebral edema, raised intracranial pressure, and possible cerebral hypoperfusion. These pathophysiological processes are closely linked to irreversible neuronal injury and increased mortality [24]. Thus, level of consciousness at admission serves as an important clinical marker of disease severity and should prompt aggressive monitoring and supportive care.

Incomplete or unknown vaccination status was another independent predictor of unfavourable outcome. Similar associations have been reported in Bahirdar [11] and Wolaita Sodo [10]. Vaccination against Haemophilus influenzae type b and Streptococcus pneumoniae has significantly reduced the incidence and severity of bacterial meningitis globally, particularly in settings with high coverage [16]. Children who are not fully vaccinated remain vulnerable to invasive strains that may cause more severe disease and complications. This finding highlights the continuing importance of strengthening immunization programs to improve meningitis outcomes.

Aspiration pneumonia was independently associated with poor prognosis. Neurological impairment in meningitis can compromise airway protective reflexes, increasing the risk of aspiration. Secondary pulmonary infection may lead to hypoxia and systemic inflammatory stress, further exacerbating cerebral injury [25]. This interaction between primary CNS infection and respiratory complications emphasizes the need for vigilant airway management and early recognition of aspiration risk in hospitalized children with meningitis.

Although adjunctive corticosteroid therapy has been evaluated in pediatric meningitis, evidence from low- and middle-income countries has been inconsistent, with limited mortality benefit reported in several African settings [24, 25]. Likewise, Randomized trials and meta‑analyses comparing shorter (≈ 5–7 days) versus longer (≈ 10–14 days) courses of ceftriaxone in pediatric bacterial meningitis have generally shown similar outcomes in children who are clinically stable by day five, although data remain limited and pathogen‑specific interpretations are not well characterized [26, 27]. In our study, all patients were managed according to standard national guidelines; however, pathogen-specific outcome analysis was not possible due to lack of culture-positive isolates. This limits interpretation of organism-specific prognosis, particularly for pathogens such as pneumococcus or Gram-negative organisms, which are often associated with higher complication rates.

This study has some limitations. Its retrospective design relied on medical record documentation, which limited availability of some clinical and laboratory variables. No causative organisms were identified in CSF cultures, precluding pathogen-outcome analysis. This may be partly due to prior antibiotic use before hospital presentation, which likely reduced culture yield and limited organism-specific analysis.Additionally, outcome assessment was limited to hospital discharge; longer-term neurological sequelae and post-discharge mortality were not evaluated. Despite these limitations, the study provides valuable insight into modifiable and severity-related predictors of short-term outcomes in a tertiary Ethiopian setting.

Overall, the findings suggest that improving early presentation, strengthening vaccination coverage, addressing childhood malnutrition, and ensuring close monitoring of children presenting with altered consciousness may contribute to better clinical outcomes in pediatric bacterial meningitis in similar resource-limited contexts.

## Conclusion

Unfavorable short-term outcomes were observed in a considerable proportion of children hospitalized with pyogenic meningitis. Malnutrition, incomplete or unknown vaccination status, aspiration pneumonia, and coma at presentation were independently associated with unfavorable outcomes. These findings underscore the need to strengthen routine immunization coverage, improve childhood nutritional status, promote early presentation to care, and ensure close monitoring of children presenting with severe neurological impairment. Addressing these modifiable and severity-related factors may contribute to improved in-hospital outcomes in similar resource-limited settings.

## Supplementary Information


Supplementary Material 1.


## Data Availability

No datasets were generated or analysed during the current study.

## Abbreviations

ABM: Acute Bacterial Meningitis
HUCSH: Hawassa University Comprehensive Specialized Hospital
ICP: Intracranial Pressure
AOR: Adjusted Odds Ratio
COR: Crude Odds Ratio
CI: Confidence Interval
IRB: Institutional Review Board
SD: Standard Deviation
HAI: Hospital Acquired Infection
DAMA: Discharge Against Medical Advice
