# Supplementary material for: Treatment outcome and associated factors of pyogenic meningitis among children at Hawassa University comprehensive specialized hospital: a retrospective cross-sectional study
Source: BMC Pediatr. 2026 Jun 18;26:810. doi: 10.1186/s12887-026-07092-y (PMC13523353; doi:10.1186/s12887-026-07092-y)
Supplement: Supplementary file 1 [file 12887_2026_7092_MOESM1_ESM.pdf]

ANNEX

sociodemographic characteristics of patient

1	Gender	1. ☐ Male<br>2. ☐ Female
2	Age of child	____________
3	Area of residence	1. ☐ Urban<br>2. ☐ Rural

Disease and Health related patient characteristics

1	Immunization status	1. ☐ Fully vaccinated.<br>2. ☐ Partially vaccinated.<br>3. ☐ Unvaccinated
2	Nutritional Status	1. ☐ Normal<br>2. ☐ Malnourished
3	Comorbidity	1. ☐ Yes<br>2. ☐ No
4	if yes to above question, what is the associated comorbidity	1. ☐ Malaria<br>2. ☐ Pneumonia<br>3. ☐ RVI<br>4. ☐ Sepsis<br>5. ☐ Other
5	Duration of illness before presentation	__________________
6	Clinical presentation & severity of illness	1. ☐ Altered mental status (GCS >8)<br>2. ☐ Coma (GCS <8)<br>3. ☐ Focal neurologic deficit (paresis, CN palsy)<br>4. ☐ signs of increased ICP<br>5. ☐ Seizures

		6. □ Headache<br>7. □ Fever (> 38 0C)<br>8. □ Positive meningeal sign

Investigations

1	CSF analysis done	1. □ Yes<br>2. □ No
2	if yes to above question,	1. □ WBC count ________<br>2. □ Neutrophil %_______<br>3. □ Protein level________<br>4. □ Glucose level________
3	CSF culture sent	1. □ Yes<br>2. □ No
4	if yes to above question, what was the culture growth	_______________________
5	Is neuroimaging done	1. □ Yes<br>2. □ No
6	if yes to above question, what was the finding	_________________________

Treatment

1	Antibiotics used	1. □ ceftriaxone /Ceftazidime<br>2. □ vancomycin<br>3. □ meropenem<br>4. □ penicillin<br>5. □ Ampicillin<br>6. □ Other
2	Corticosteroid use	1. □ Yes<br>2. □ No

3	ICP management	1. ☐ Yes<br>2. ☐ No
4.	Anticonvulsant use	1. ☐ Yes<br>2. ☐ No

Treatment outcome upon discharge

1	**Treatment outcome,** mark one	1. ☐ Cured and Discharged<br>2. ☐ Developed Acute neurologic complication. If yes specify________<br>3. ☐ self-discharged against medical advice<br>4. ☐ Death
